# Appropriateness of Using Patient-Derived Xenograft Models for Pharmacologic Evaluation of Novel Therapies for Esophageal/Gastro-Esophageal Junction Cancers

**DOI:** 10.1371/journal.pone.0121872

**Published:** 2015-03-31

**Authors:** Lorin Dodbiba, Jennifer Teichman, Andrew Fleet, Henry Thai, Maud H. W. Starmans, Roya Navab, Zhuo Chen, Hala Girgis, Lawson Eng, Osvaldo Espin-Garcia, Xiaowei Shen, Bizhan Bandarchi, Joerg Schwock, Ming-Sound Tsao, Hala El-Zimaity, Sandy D. Der, Wei Xu, Robert G. Bristow, Gail E. Darling, Paul C. Boutros, Laurie E. Ailles, Geoffrey Liu

**Affiliations:** 1 Department of Medical Biophysics, University of Toronto, Toronto, ON, Canada; 2 Wayne State University, School of Medicine, Detroit, Michigan, United States of America; 3 Ontario Cancer Institute, Toronto, ON, Canada; 4 University Health Network, Princess Margaret Hospital, Toronto, ON, Canada; 5 Informatics and Biocomputing Program, Ontario Institute for Cancer Research, Toronto, Canada; 6 Department of Radiation Oncology (Maastro), GROW-School for Oncology and Developmental Biology, Maastricht University Medical Center, Maastricht, Netherlands; 7 Department of Anatomical Pathology, Toronto General Hospital, Toronto, ON, Canada; 8 Division of Thoracic Surgery, Toronto General Hospital, Toronto, ON, Canada; 9 Division of Epidemiology, Dalla Lana School of Public Health, Toronto, Canada; 10 Department of Biostatistics, Princess Margaret Hospital, Toronto, Canada; 11 Department of Pharmacology & Toxicology, University of Toronto, Toronto, ON, Canada; Peter MacCallum Cancer Centre, AUSTRALIA

## Abstract

The high morbidity and mortality of patients with esophageal (E) and gastro-esophageal junction (GEJ) cancers, warrants new pre-clinical models for drug testing. The utility of primary tumor xenografts (PTXGs) as pre-clinical models was assessed. Clinicopathological, immunohistochemical markers (p53, p16, Ki-67, Her-2/*neu* and EGFR), and global mRNA abundance profiles were evaluated to determine selection biases of samples implanted or engrafted, compared with the underlying population. Nine primary E/GEJ adenocarcinoma xenograft lines were further characterized for the spectrum and stability of gene/protein expression over passages. Seven primary esophageal adenocarcinoma xenograft lines were treated with individual or combination chemotherapy. Tumors that were implanted (n=55) in NOD/SCID mice had features suggestive of more aggressive biology than tumors that were never implanted (n=32). Of those implanted, 21/55 engrafted; engraftment was associated with poorly differentiated tumors (p=0.04) and older patients (p=0.01). Expression of immunohistochemical markers were similar between patient sample and corresponding xenograft. mRNA differences observed between patient tumors and first passage xenografts were largely due to loss of human stroma in xenografts. mRNA patterns of early vs late passage xenografts and of small vs large tumors of the same passage were similar. Complete resistance was present in 2/7 xenografts while the remaining tumors showed varying degrees of sensitivity, that remained constant across passages. Because of their ability to recapitulate primary tumor characteristics during engraftment and across serial passaging, PTXGs can be useful clinical systems for assessment of drug sensitivity of human E/GEJ cancers.

## Introduction

In recent decades, esophageal (E) and gastro-esophageal junction (GEJ) cancer has seen a dramatic rise in incidence in developed countries while the five-year survival remains low at 19% [1]. Identifying methods to select appropriate drug therapies are therefore warranted. Traditionally, cell line panels have been used to rapidly test anti-cancer agents [2,3]. In addition, injections of cell lines into immunocompromised mice are common *in vivo* models of drug efficacy [4,5]. Although cell line approaches have greatly contributed to drug development and cancer biology, they are imperfect models for drug testing [6]. Furthermore, three widely used E/GEJ cancer cell lines were recently found to have been contaminated by other cell lines early in culture [7]. Thus, idetifying appropriate pre-clinical drug-testing models of this cancer is a challenge.

Primary tumor xenografts (PTXGs) show promise as alternative pre-clinical models for drug sensitivity testing. PTXGs are created by implanting a piece of tumor directly from a patient into immunocompromised mice and using the resultant xenograft for experimentation. Primary tumor xenograft models of lung [8], breast [9], colon [10], head and neck [11] and E/GEJ cancers [12] have been shown to recapitulate the patient tumor histology and cell morphology to varying degrees. Physiological conditions such as temperature, oxygen levels, nutrient content *etc*. more closely resemble those present in cancer patients [6]. In addition, PTXGs have not undergone the selection pressures and significant molecular changes involved in cell line establishment and long-term growth [6], [13,14]. Thus, PTXGs might be more likely to predict drug responses than cell lines grown either *in vitro* or *in vivo*. However, while PTXGs have been shown to mimic the original tumor response to treatment quite accurately [15–17], they are still exposed to selection forces related to differences between human and mouse microenvironments [18]. Thus it is important to assess the degree to which PTXGs diverge from the primary patient tumors at the genomic and protein level prior to embarking on extensive drug studies.

We have previously described PTXG model development [12]. The next logical step is the overarching goal of the present study: to assess the utility and limitations of using PTXG models for drug testing. Specifically, to what degree are E/GEJ tumors representative of patient tumors, in the context of pharmacologic evaluation? Our own experiments across multiple cancer types have identified common PTXG issues such as: (i) lack of engraftment of many tumors; (ii) unexpected mouse deaths, leading to the need to alter and run experiments across different passages; (iii) technical reasons, such as the need to expand xenografts into large cohorts and to freeze down earlier passages to ensure accessibility for future studies, resulting in having to use later passages for drug experiments; and (iv) an occasional inability to identify specific PTXG models with the same molecular characteristics observed in a patient.

These technical issues raise four important model-related questions, which can be stated as testable hypotheses: (a) PTXG models are representative of the underlying population of gastro-esophageal cancers (i.e., assessing engrafting bias), and if not wholly representative, we are able to describe what biases are present and how they could affect conclusions; (b) PTXG models are useful generally, even at later passages, as their gene/protein expression patterns remain stable; this hypothesis measures the degree of confounding by selection pressures during passaging; (c) PTXGs can recapitulate the broad spectrum of molecular characteristics representative of their underlying primary cancers; this addresses the concerns of not finding the inter-tumor heterogeneity necessary to develop personalized medicine approaches to therapy; and (d) PTXGs respond to pharmacologic therapy stably across multiple passages. Thus, we evaluated the potential of E/GEJ PTXGs to replicate what is found clinically in human E/GEJ tumors, and the conditions in which these PTXGs are appropriate to use as pre-clinical drug testing models.

## Materials and Methods

### Clinical and Pathological Patient Information

All patients were recruited through written consent. Patient information, including treatment and outcome (*i*.*e*. overall survival) was obtained through the Electronic Patient Records system at the University Health Network (UHN), Toronto, Canada, by gastro-intestinal oncologists. The UHN Research Ethics Board (REB #: 06-0982-CE) approved the study.

### Animals and Tissue Processing

Non-Obese Diabetic-Severe Combined Immune Deficient (NOD/SCID) mice were bred internally at the Ontario Cancer Institute (OCI) Animal Care facility. All animals were kept in a pathogen free environment on a standard 12hr-day/12hr-night cycle and were fed a standard sterilized pellet diet and water *ad libitum*. Animals were sacrificed using carbon dioxide followed by cervical dislocation. All procedures were approved by the ethical guidelines of the OCI Animal Care Committee (animal protocol #: 1293.16).

Human E/GEJ cancer tissues were obtained from patients undergoing surgical resection at UHN, Toronto, Ontario and were processed as previously described [12]. In summary, fresh tissue was stored in RPMI 1640 medium (no FBS added) until it was cut into pieces of approximately 5mm dimensions and implanted subcutaneously into the flank of NOD/SCID mice (within 24 hours of resection; however, cancer tissues were placed in tissue culture media within a median of 45 minutes after resection, and kept in media until implantation). Two representative pieces were saved in Optimal Cutting Temperature (OCT) compound (frozen at -80°C for molecular work) and formalin fixed paraffin embedded (FFPE) blocks for molecular and pathological analysis, respectively.

### Treatment

Cohorts of 10 implanted mice per treatment arm were monitored for tumor growth. Once visible, tumors were measured using metric calipers (Scienceware, cat: 134160001) twice weekly. Mice were sacrificed when tumors reached 15–20 mm on the longest dimension, considered a humane endpoint by the Animal Care Committee. Once tumors reached approximately 300–750 mm^3^, mice were randomized into control and chemotherapy arms. For single treatment experiments, mouse cohorts were injected once intraperitoneally with 6 mg/kg of cisplatin (1mg/mL, Hospira, DIN:02126613), 9 mg/kg of paclitaxel (2mg/mL, Hospira, DIN:02296624) or 100 mg/kg of 5-fluorouracil (15mg/mL, Hospira, DIN:021827420) while the control group was injected with saline. For combined treatment experiments, the chemotherapy group was treated once with 5.4mg/kg of cisplatin (1mg/mL, Hospira, DIN:02126613) and 9 mg/kg of paclitaxel (2mg/mL, Hospira, DIN:02296624), based on mouse toxicity experiments. Mice were sacrificed 24 hours after the initial treatment (labeled as small tumors) and at the end of the experiment (large tumors). A median of two mice (1–3) per arm were sacrificed for comparisons. Tumors were harvested and saved in OCT and FFPE blocks for molecular/pathological characterization.

### Immunohistochemistry of Molecular Markers

We evaluated a panel of molecular markers by immunohistochemistry (IHC) to study their stability within each xenograft. Markers were chosen based on their importance to E/GEJ cancer. FFPE tissue sections were stained with antibodies against p53 (clone DO-7, Vector Laboratories, Burlington,Canada, dilution:1:250), p16^INK4a^ (mtm laboratories AG, Heidelberg,Germany; not- diluted), Ki-67 (Clone SP6, NEOmarkers, Rockford,USA, dilution:1:500), EGFR (Clone 31G7, Zymed, San Fransisco,USA, dilution:1:100), Her-2/*neu* (clone A0485, Dako, Burlington,Canada, dilution:1:25), HIF-1α (clone 54, BD Biosciences, Franklin Lakes,USA, dilution:1:50) and CD31 (clone PECAM1, Santa Cruz,USA, dilution:1:1000). A staining index was used to evaluate CD31 and HIF-1α expression using Aperio ImageScope viewer. All other molecular markers were evaluated by a blinded team of pathologists. A combination of a proportion score and an intensity score was used. The proportion score (proportion of positive tumor cells) was: 0: none, 1: 1–24%, 2: 25–49%, 3: 50–74, 4: ≥75%. The intensity score (intensity of staining by tumor cells) was: 0: none, 1: weak, 2: moderate, 3: strong. A total score was obtained by combining both scores. Briefly, p53 and p16 were considered positive if staining appeared while Ki-67 was evaluated based on the percentage of positive tumor cells. EGFR membrane expression determined EGFR-positivity. Her-2/*neu* standard scoring was used; samples were considered to have similar expression if they differed by less than one stain intensity score (eg. 2+ vs 3+). Two sided t-tests were used to evaluate differences in expression for these parameters.

### RNA Extraction

RNA was extracted from OCT embedded xenograft and human tissue (ten x 10μm thick cryostat slices) using RNeasy Mini-kits (Qiagen,74104). RNA integrity was assessed using the Agilent-2100-Bioanalyzer and the RNA Nano-6000-kit (Agilent Technologies,5067–1511) and quantified using a Nanodrop-ND-1000 Spectrophotometer (Thermo-Scientific). RNA integrity numbers (RIN) of 7+ or 8+ were as quality cutoffs for patient and PTXG samples, respectively.

### Labeling and Hybridization

Samples were labeled according the GeneChip WT Terminal Labeling and Hybridization manual, hybridized to Human Gene-1.1-ST arrays (Affymetrix) and scanned with the GeneChip Scanner 3000-7G (Affymetrix). All arrays passed quality control criteria (Expression Console software, Affymetrix).

### Statistical Analysis

For chemosensitivity analysis, the delay in time for a tumor to double in volume was compared between treated lines and their corresponding untreated controls. Time delays were determined by plotting the tumor volumes on a logarithmic y-scale and drawing a horizontal line at the doubling volume that intercepted the control and treatment growth curves. The time delay was determined as the difference in time between the two intercepts and was referred to as the doubling time delay. The average doubling time delay for resistant vs. sensitive lines was calculated by pooling all control mice and comparing them with the pooled values of treated mice.

Additional statistical analyses were performed on clinicopathological factors to determine bias in the study sample, and to determine factors associated with engraftment. Two-sided t-tests were applied in all cases. Results were considered significant when *p*-value<0.05. Statistical analyses were performed using SAS.v.9.2 (Cary,NC).

### Gene Profiling Analysis

Both primary patient tumor and matched xenograft samples were used for transcriptome analyses (in total, 51 samples were analyzed for mRNA abundance). Twenty-one adenocarcinoma tumors were profiled (12 engrafted/9 non-engrafted). For eight engrafted primary adenocarcinomas, a passage one (P1) xenograft sample was profiled. In addition, analysis was performed on 21 later passage xenografts (P3 through P12), of which nine were from small (early in the growth curve) tumors and 12 were from large tumors.

We assessed differences in mRNA profiles between (a) patient tumors that engrafted *vs* those that did not, (b) P1 xenografts *vs* P0 (primary) tumors, (c) later passage xenografts *vs* P1, and (d) large *vs* small xenograft tumors within the same passage. For each comparison, a gene-wise linear model was fit to compare the two conditions, adjusted for array batch and, where appropriate, for passage. A false-discovery rate correction was performed to account for multiple testing [19].

All analyses were performed using R(v2.15.3). The packages lattice (v0.20–15) and latticeExtra (v0.6–24) were used for graphical representation. Data pre-processing was performed using the RMA algorithm [20] (Affymetrix package,v1.36.0) combined with updated ProbeSet annotations (hugene11stv1hsentrezgcdf package,v15.0.0) [21]. An expression filter was used to remove experimental noise. A low-intensity threshold was set based on the chromosome-Y gene intensities of the five female patient samples as described previously [22]. Probes with a mean log_2_-transformed expression value below five were removed. In total, ~15,500 probes were retained.

A power analysis was performed with the power.t.test function (stats package v.2.15.3) to estimate the likelihood that differences in expression levels could be identified between groups. Since the expression data was in log_2_-space, a difference in mean expression was equivalent to the log_2_-transformed fold-change. The number of observations per group was set to 10 and the significance level was set at 0.05/15,500 = 3x10^-6^ (*i*.*e*., Bonferroni corrected). Power was calculated for a range of fold-changes (|log2 fold-changes| from 1.1 to 6) and of standard deviations. We calculated the distribution of standard deviations for gene expression and calculated power for all deciles. For 50% of the genes, there was an 80% chance of detecting a |log_2_ fold-changes| of 1.7 (~ FC 3) and higher.

## Results

### Clinico-Molecular Characteristics Associated With Implantation/ Engraftment

**Fig 1:**
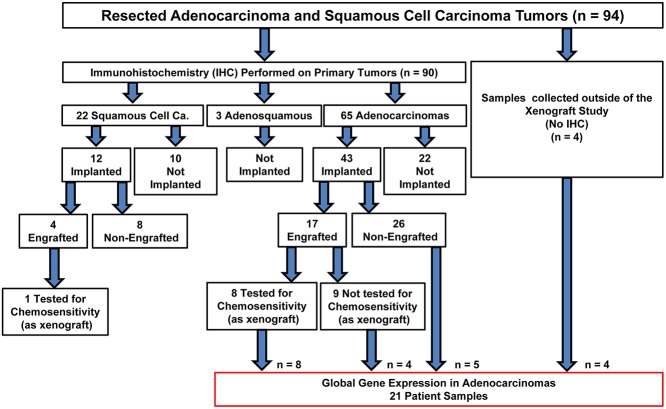
Consort diagram describing patient tumors used in this study, including those evaluated for global mRNA abundance, the expression of molecular markers through immunohistochemistry (IHC), and chemosensitivity as patient-derived xenografts.

Key molecular markers that reflect important pathways relevant to E/GEJ were evaluated in 90 primary resected tumors, 55 of which had sufficient tissue at resection to be implanted (Fig 1). Of implanted samples, 21 (38%) engrafted successfully (4 squamous cell carcinomas (SCC); 17 adenocarcinomas).

Since not all resected tissues were available for implantation, it was important to determine if any biases exist between implanted and non-implanted specimens. A multivariate analysis of both subtypes (SCC, adenocarcinoma) and of adenocarcinoma only, revealed a higher likelihood of implantation with tumors located in the GEJ (compared with either Lower Third/Distal or Mid/Upper tumors, p = 0.01; Table 1).

**Table 1:** Multivariate analysis of Clinicopathological and Immunohistochemical Characteristics of Patient Tumors.

**Outcome**	**Characteristics**	**Comparison**	**N**	**Y**	**OR (95% CI)**	**p-value**
		GE Junction	9	32	Reference	
**Implanted (Y) *vs* Non-Implanted (N) (All Tumors)**	Location	Lower Third/Distal	15	13	0.17 (0.05,0.54)	0.01
		Mid/Upper	4	6	0.28 (0.06,1.35)	
	Heartburn	No or n/a	19	28	Reference	0.05
		Yes	9	23	3.12 (1.01,9.69)	

	Age (years)	*Per 10 Year Increase	n/a		1.10 (1.02,1.18)	0.01
**Engrafted (Y) *vs* Non-Engrafted (N) (All Tumors)**	Her-2/*neu*	Per Increase in 1 Level of Intensity Stain	n/a		1.93 (0.98,4.19)	0.1
	Differentiation	Mod/Well	24	8	Reference	0.04
		Poorly	8	11	4.41 (1.07,18.2)	

	Age (years)	*Per 10 Year Increase	n/a		1.09 (1.02,1.16)	0.01
**Engrafted (Y) *vs* Others (N) (All Tumors)**	Her-2/*neu*	Per Increase in 1 Level of Intensity Stain	n/a		2.38 (1.09,5.17)	0.03
	Differentiation	Mod/Well	42	8	Reference	0.01
		Poorly	18	11	6.48 (1.63,25.8)	
		GE Junction	28	13	Reference	

	Location	Lower Third/Distal	25	3	0.14 (0.03,0.79)	0.06
		Mid/Upper	7	3	1.93 (0.32,11.5)	
	Location	GE Junction	9	31	Reference	0.003
		Lower Third/Distal	12	8	0.11 (0.03,0.47)	
**Implanted (Y) *vs* Non-Implanted (N) (Adenocarcinomas)**	Heartburn	No or n/a	15	23	Reference	0.06
		Yes	6	16	4.20 (0.92,17.6)	

	Age	*Per 10 Year Increase	n/a		1.08 (1.00,1.17)	0.04
**Engrafted (Y) *vs* Non-Engrafted (N) (Adenocarcinomas)**	Her-2/*neu*	Per Increase in 1 Level of Intensity Stain	n/a		2.51 (0.99,6.38)	0.05
	Differentiation	Mod/Well	18	5	Reference	0.02
		Poorly	6	10	9.90 (1.54,63.8)	

	Age (years)	*Per 10 Year Increase	n/a		1.09 (1.01,1.18)	0.02
**Engrafted (Y) *vs* Others (N) (Adenocarcinomas)**	Her-2/*neu*	Per Increase in 1 Level of Intensity Stain	n/a		3.48 (1.30,9.33)	0.01
	Differentiation	Mod/Well	31	5	Reference	0.01
		Poorly	14	10	13.2 (2.11,82.1)	
	Location	GE Junction	27	13	Reference	0.02
		Lower Third/Distal	18	2	0.05 (0.00,0.60)	

The final multivariate models are shown. Factors assessed included age, gender, stage, differentiation, location, neo-adjuvant chemo-radiation, heartburn, Barrett’s esophagus and expression of p16, p53, Her-2/*neu*, EGFR and Ki-67.

*Age was modeled as a continuous variable in the logistic regression analysis; the odds ratio is reported for every increase in 10 years. For example, this is the odds ratio comparing someone aged 70 vs 60 years old; or 65 vs 55 years old.

Of implanted samples, successful engraftment was higher in tumors coming from older patients (OR = 1.10/ten year increase, p = 0.01) and in poorly differentiated tumors (OR = 4.41, p = 0.04). Her-2/*neu* expression was significantly higher in engrafting adenocarcinoma specimens (OR = 2.51, p = 0.05). A comparison between engrafted and all other specimens (non-engrafted and non-implanted) showed that tumors from older patients (OR:1.09, p = 0.01), Her-2/*neu* positivity (OR = 2.38, p = 0.03), and poor differentiation (OR = 6.48, p = 0.01) were each associated with a higher chance of engraftment. The same comparison revealed that in the adenocarcinoma subset, the same variables, in addition to GEJ adenocarcinomas, were associated with higher engraftment (p = 0.02; Table 1). A Cox proportional hazard model revealed that overall survival was not significantly different by engraftment or implantation status (p>0.05). Univariate analyses are shown in S1 and S2 Tables.

A global mRNA abundance analysis of engrafted (n = 12) and non-engrafted (n = 9) adenocarcinoma samples did not reveal any differences between the two groups. Even without adjusting for multiple testing, only 76 genes across a variety of different pathways showed a difference in expression with p < 0.01.

### Comparison of First Passage (P1) Xenografts vs Primary Tumors

**Fig 2:**
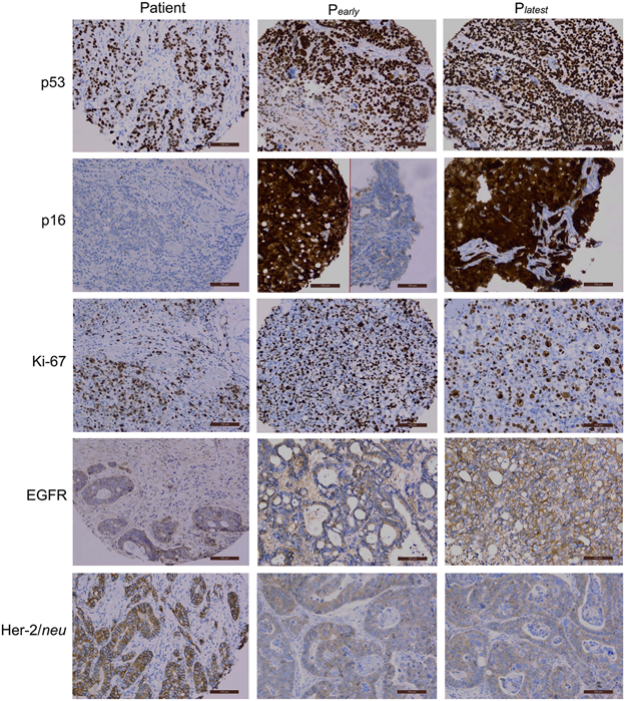
Selected molecular marker expression by immunohistochemistry (IHC). P53 in Line A and Ki-67 in Line H are examples of similar expression between patient, early passage (P1) and latest passage (P_*latest*_) xenografts. P16 in Line H was selected to demonstrate the heterogeneity detected in the same tissue (P_*early*_ showing both positive and negative expression). EGFR expression in Line E exhibited an increase in intensity from patient to xenografts while Her-2/*neu* expression in Line A showed a decrease in intensity. These examples were included to demonstrate that the differences exhibited between patient tissue, early passage and latest passage xenografts were due to intrinsic heterogeneity and not to any specific patterns of expression.

To determine molecular marker changes with engraftment, protein expression was evaluated in seven pairs of patient tumors and corresponding xenografts (Table 2A). P53, p16 and EGFR were scored as “matching” or not, as these were typically on/off phenomena, without wide variation in the degree of positivity. P53 (5 of 7 cases had primary-xenograft match), p16 (6/7), Her-2/*neu* (6/7), and EGFR membrane expression (5/7) showed high concordance between patient tumors (P0) and early passage (P1) xenografts. Ki-67 indices were also similar between patient tumors and xenografts (p>0.05). Expression heterogeneity was present in the remaining cases. Examples of the expression of these markers between patient tumors and early xenografts are shown in Fig 2.

**Table 2:** Comparison of immunohistochemical (IHC) staining patterns between primary human adenocarcinoma and xenograft (A), early and late xenograft passages (B), and small and large tumors within the same passage (C).

**Line**	**A—Primary Human Tumor (H) *vs* Xenograft (X)**					**B—P1 (E) *vs* P**_latest_ **(L) Xenografts**					**C—Small (S) *vs* Large (L) Tumors**				
	**p53**	**p16**	**Ki-67% (+)ve**	**EGFR MembraneStatus**	**Her-2/*neu*Score** *	**p53**	**p16**	**Ki-67% (+)ve**	**EGFRMembraneStatus**	**Her-2/*neu*Score** *	**p53**	**p16**	**Ki-67% (+)ve**	**EGFRMembraneStatus**	**Her-2/*neu* Score** *
A	H+	H-	H = 75	H+	H = 3+	E+	E+	E = 75	E+	E = 3+	S+	S-	S = 45	S+	S = 1+
	X+	X-	X = 75	X+	X = 3+	L+	L+	L = 53	L+	L = 2+	L+	L-	L = 53	L+	L = 2+
B	H-	H-	H = 6	H-	**H = 1+**	E-	E-	E = 55	E-	E = 3+	S-	S-	S = 55	S-	S = 3+
	X-	X-	X = 55	X-	**X = 3+**	L-	L-	L = 40	L-	L = 3+	L-	L-	L = 40	L-	L = 3+
C	H-	H-	H = 42	**H -**	H = 2+/3+	E-	E-	E = 55	E+	E = 3+	S-	S-	S = 85	S+	S = 3+
	X-	X-	X = 55	**X +**	X = 3+	L-	L-	L = 60	L+	L = 3+	L-	L-	L = 60	L+	L = 3+
D	n/a	n/a	n/a	n/a	n/a	E-	E-	E = 75	**E-**	**E = 1+**	S-	S-	S = 60	S+	S = 3+
	n/a	n/a	n/a	n/a	n/a	L-	L-	L = 65	**L+**	**L = 3+**	L-	L-	L = 65	L+	L = 3+
E	H-	H-	H = 65	H +/-	H = 0	E-	E-	E = 45	E+	E = 0	S-	S-	S = 85	S+	S = 1+
	X-	X-	X = 45	X -	X = 0	L-	L-	L = 88	L+	L = 1+	L-	L-	L = 88	L+	L = 1+
F	n/a	n/a	n/a	n/a	n/a	n/a	n/a	n/a	n/a	n/a	S-	S+	S = 55	**S+**	S = 2+
	n/a	n/a	n/a	n/a	n/a	n/a	n/a	n/a	n/a	n/a	L-	L+	L = 25	**L -**	L = 1+
G	**H +**	H-	H = 60	H-	H = 0/1+	**E-**	E-	E = 75	E-	E = 1+	S+	S-	S = 48	S-	S = 0
	**X -**	X-	X = 75	X-	X = 1+	**L+**	L-	L = 38	L-	L = 1+	L+	L-	L = 38	L-	L = 1+
H	H-	**H -**	H = 47	H-	H = 0	**E-**	E-/+	E = 50	E-	E = 1+	n/a	n/a	n/a	n/a	n/a
	X-	**X+/-**	X = 50	X-	X = 1+	**L+**	L+	L = 80	L+	L = 1+	n/a	n/a	n/a	n/a	n/a
I	H +/-	H-	H = 24	H-	H = 0	n/a	n/a	n/a	n/a	n/a	n/a	n/a	n/a	n/a	n/a
	X+	X-	X = 80	X-	X = 0	n/a	n/a	n/a	n/a	n/a	n/a	n/a	n/a	n/a	n/a

* A difference intensity of less than or equal to 1 is considered a match.

Bolded data highlight mis-match

(-) Indicates negative staining; (+) indicates positive staining; for Her2/*neu* staining a score was assigned from 0 to 3+ based on proportion of cells stained combined with staining intensity.

n/a—Tissue not available for comparison.

**Fig 3:**
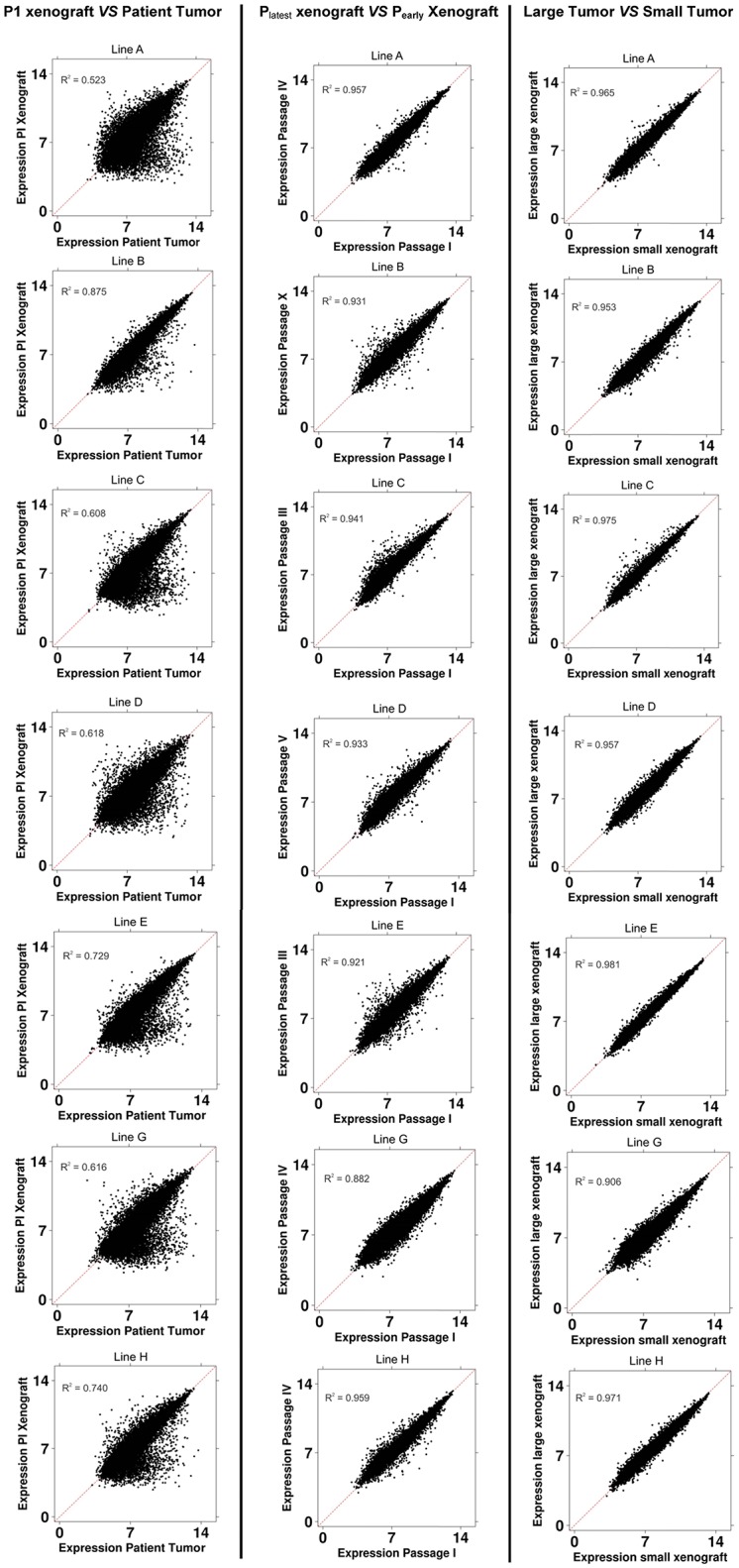
Scatterplot showing mRNA abundance comparisons for each established adenocarcinoma line. Comparisons were made between P1 xenograft *vs* patient tumor (left column), P_latest_ *vs* P_early_ xenograft (middle column) and Large *vs* Small xenograft tumors (right column). Normalized expression levels for individual genes were used to plot the comparison. R^2^ values are included for each comparison. mRNA for lines F and I could not be extracted for all comparisons since mRNA degradation in the frozen tissue had occurred. Both samples had intact mRNA for the patient tumor but Line I did not have a matching later passage xenograft while Line F did not have a matching first passage xenograft. Both lines were included in statistical comparisons where the data was present.

In the mRNA abundance analysis of primary patient tissue *vs* first passage xenografts, we found good concordance (Fig 3, first column) with R^2^ values ranging from 0.52–0.88. Unsupervised hierarchical clustering showed that xenografts clustered together while patient tumors clustered with other patient tumors (S1 Fig). 2164 genes were differentially expressed between P0 and P1 tumors (false discovery rate <5%).

A list of GO (gene ontology) terms was used to verify that the most significant mRNA abundance differences between P0 and P1 are due to loss of human stroma in the xenograft. Terms related to key words such as immune system process, endothelial cell differentiation, fibroblast proliferation, erythrocyte differentiation *etc*. were used as selective markers for stromal genes. We used a stringent definition for inclusion as a stromal gene, with the expectation that many more potential stromal genes may be missed. Using this definition, of the 15,630 genes on the microarray, 1,410 (9.02%) were related to stroma. Of the 2,164 genes that showed significant differential expression between primary tumors and passage I xenografts 338 (15.6%) were related to stroma, demonstrating enrichment (p = 0.0001).

### Changes Occurring After Serial Passaging

To assess whether significant molecular changes occur in xenografts after multiple passages, we evaluated the same molecular markers as above in early passage (P1, large tumors) and late passage (latest passage available for comparison, large tumors) for seven lines. Similarly to the previous analysis, p53 (5 of 7 were similar), p16 (6/7), Her-2/*neu* (6/7), EGFR membranous expression (5/7) and Ki-67 index showed good correlation between early and latest passage xenografts (Table 2B). Examples of the expression of these markers between early and latest passage xenografts are shown in Fig 2.

Early and late passage xenograft mRNA abundance profiles showed that no significant differences remained after false discovery rate adjustment. In a prospective subanalysis using a less stringent threshold for significance (p<0.01), only 64 genes showed a difference in expression. In addition, scatterplots of the gene expression in early passage (P_early_) and latest passage (P_latest_) of each xenograft line showed that there were very few changes occurring as the xenografts were passaged serially (Fig 3, second column). A hierarchical clustering analysis found that xenografts clustered within their corresponding tumor lines and not according to passage (S1 Fig).

### Small and Large Xenografts within the Same Passage

Small (early in the growth curve) and large (late in the growth curve) tumors within the same passage were also compared to determine if there were any underlying differences in xenografts within the same passage (but of different sizes). P53, p16, Her-2/*neu* and EGFR expression were similar (Table 2C). mRNA abundance analysis showed that no differentially expressed genes could be identified after false discovery rate adjustment. Only 23 genes were differentially expressed when using a less stringent threshold for significance (p<0.01).

### Tumor Growth Kinetics and Chemosensitivity

**Fig 4:**
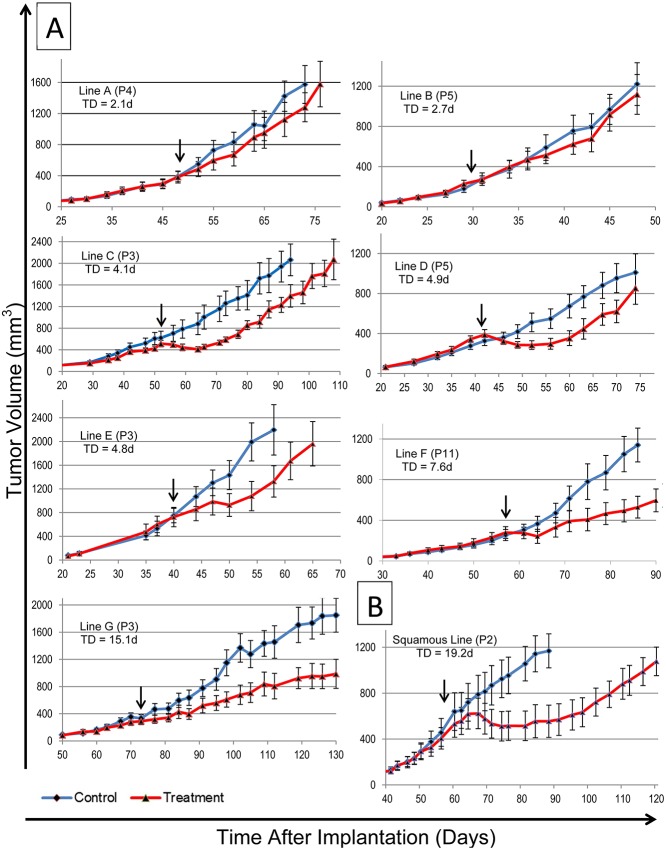
Chemosensitivity of primary E/GEJ cancer xenograft lines was tested with a combined dose of paclitaxel and cisplatin. Means ± SEM (n = 10 mice per group) were plotted. Arrows indicate the time at treatment. Lines are arranged in ascending order of their time delay at doubling volume (TD), measured in days (d). (**A**) Seven adenocarcinoma lines range in the degree of sensitivity to these agents. Lines A and B show clear signs of resistance while the remaining lines show various degrees of sensitivity. Line H was tested for chemotherapy but due to mouse toxicity could not be completed. Due to the slow growing nature of the tumor, Line I was not tested because not enough tissue could be propagated. (**B**) The squamous cell carcinoma line is very sensitive to the treatment and is presented here for comparison purposes. Note: The data for Line B (P5) and Line F (P11) has previously been published and corresponds to Line 8 (P5) and Line 1 (P11) in Dodbiba et al. Lab. Invest. 93, 397–407 (2013).

To determine whether E/GEJ xenografts can be used to assess drug responses (sensitivity and resistance), we treated and analyzed the response of multiple lines to chemotherapy. Cisplatin, paclitaxel and 5FU were chosen because of their extensive use in clinical settings for E/GEJ cancer cases. Initial results with two lines (lines B and F in Fig 4) were previously reported in Dodbiba et al, 2013[12]. Here we extend our studies to a larger number of xenograft lines.

Tumor lines exhibited a wide range of chemosensitivity to a combined dose of paclitaxel and cisplatin (Fig 4). When arranging the lines in ascending order of doubling time delay, the adenocarcinoma lines formed distinct patterns (S2 Fig). Resistant adenocarcinoma lines showed little to no delay (average, 2.3 days; Lines A and B), while the sensitive lines could be divided into two categories: (1) Sensitive-Trough lines show initial shrinkage after treatment, followed by regrowth at a similar rate to the control group (average doubling time delay of 4.4 days, Lines C and D); and (2) Sensitive-ΔSlope lines show no or very little shrinkage but growth proceeded with a decreased slope (representing slower growth) compared to the controls (average delay at doubling time of 6.7 days, Lines E, F and G). The largest delay (*i*.*e*., largest chemosensitivity) was exhibited by the squamous cell carcinoma line (average delay of 19.6 days).

In order to evaluate if chemosensitivity properties of the lines changed with passage, we treated three lines (B, F and D) with different chemotherapy combinations across different passages. The behavior of each line was similar between passages and across treatment modalities. Line B exhibited resistance to individual treatments of cisplatin, paclitaxel and 5FU at passage 3, and was similarly resistant to a combined dose of cisplatin and paclitaxel at passage 5 (S3 Fig). Line F showed sensitivity to initial individual treatments of cisplatin, paclitaxel and 5FU at passage 8 and gained resistance to these treatments upon being given a second dose. At passage 11, the line again exhibited sensitivity to an initial combined dose of cisplatin and paclitaxel, and similarly showed no additional growth delay upon the second treatment (S3 Fig). Chemosensitivity to the same treatment (combined dose of cisplatin and paclitaxel) did not change across different passages of Line D (S3 Fig). When analyzing the doubling time delay for these lines at different passages, chemosensitivity did not change from passage to passage. Overall, inherent resistance, gain of resistance and chemosensitivity remained constant across passages.

### Molecular Markers, Chemosensitivity and Response in Human Counterpart

Expression of p53, p16, Ki-67, EGFR and Her-2/*neu* were similar across tumors of different chemosensitivity patterns. In order to determine if hypoxia was related to chemoresistance, we also evaluated HIF-1α nuclear expression and CD31 expression in paired control and treated tumors. Neither CD31 nor HIF-1α showed any changes in expression after treatment at early growth stages or the endpoint (p>0.05). In addition, no changes in protein expression were detectable between the three main chemosensitivity categories (p>0.05), although this study was not powered to detect minor differences. Thus none of the markers tested were predictive of therapy responsiveness, but all remained constant before and after chemotherapy (data not shown).

Of seven lines that were chemo-treated as xenografts, only three were also treated using chemotherapy in their human counterparts (Line A, D and E). Of these, only two patients could be compared for treatment response with their corresponding xenografts (Lines D and E). Response to treatment in the patients was evaluated *via* a CT scan performed within two months of starting treatment. Both Patient D (treated with Epirubicin, Cisplatin and 5FU) and Patient E (5FU, Cisplatin and Radiation) had stable disease after treatment. Although the patients are treated differently, this observation is similar with what was seen in the xenografts where the sensitivity of Line D and E were found in the middle of the response chart (S2 Fig).

## Discussion

To assess the potential utility and limitations of E/GEJ PTXGs, we demonstrated the following:

- Engrafted tumors had poorer differentiation and higher stain intensity of Her-2/*neu*, compared to non-implanted and/or non-engrafted tumors. This tendency to have more biologically aggressive tumors [23–25] engrafted is useful to our goals, since the objective of pre-clinical drug testing is to develop novel therapies that target treatment refractive patients. Of note is that not all resected tissues were available for implantation because of the need to prioritize clinical use. Smaller tumors or tumors whose margin involvement was questionable or difficult to separate from scarring due to prior chemo-radiation therapy or lack of availability of a pathologist within the time frame to approve research use of the sample, affected which tissue would be available for implantation. That being said, the engrafted vs all other tumors comparison was performed to identify these potential selection biases in the tumors that were implanted, and does not reflect potential biological processes related to engraftment capabilities of the tumor. Awareness of these biases indicates that future efforts should aim at identifying methods for successful engraftment of tissues from all patients to enable studies aimed at developing or optimizing therapeutic strategies in clinically relevant models representative of all types and stages of disease.
- Minimal differences were observed in EGFR, Her-2/*neu*, Ki-67, p16 and p53 staining between patient tumors and primary xenografts, and between early and late passage xenografts, indicating stability in protein expression during engraftment and with serial passaging. Although most lines remained stable across multiple passages, a few lines showed distinct differences in the expression of specific markers in different passages, but there was no consistent pattern. Such fluctuations may be associated with a high level of heterogeneity present in patient samples that is reflected in the xenograft samples, though we cannot exclude passaging effects (*e*.*g*. selection of a subclone) in some cases.
- Another feature of these models is the heterogeneous expression of some of the markers within individual xenografts (*i*.*e*. intratumor heterogeneity). Often, samples of the same line would show great variation in the expression of a certain marker, even within tumors from the same passage. This is a potential strength of the PTXG model. Intratumor heterogeneity is well documented clinically, and can be related to genetic [26–30] and epigenetic [31,32] alterations found in different areas of a tumor. Thus, the number of mice needed to study responses to treatment must be large enough to account for regional differences within tumors and for sampling variation. This also has repercussions in the discovery and application of predictive and prognostic biomarkers: inadequate tumor sampling could lead to suboptimal therapeutic decision making. Importantly, this heterogeneity seems to be well conserved in E/GEJ cancer xenografts, although formal assessment of clonal heterogeneity within patient samples and xenografts remains to be done. Indeed interrogation of clonal heterogeneity in patient tissues and xenografts is currently a major focus of study in the cancer research community. However, this requires a deep analysis of patient vs. xenograft genomic DNA for the identification and quantification subclones and is beyond the scope of the current study.
- Through mRNA abundance profiling, the most significant global differences were seen during xenograft establishment (P1 *vs* P0), but these differences were mainly due to mouse stroma replacing human stroma, a phenomenon documented in other xenograft models [33]. Nonetheless, the vast majority of expressed genes remained relatively stable between primary tumors and early passage xenografts, and the segregation of xenografts and patient tumors upon unsupervised hierarchical clustering (S1 Fig) is most likely driven primarily by the loss of stromal genes. mRNA abundance profiling also demonstrated an overall stability both over tumor growth (small *vs* large tumors within the same passage) and over serial passage (P_early_ *vs* P_late_).
- We also described the growth kinetics and chemosensitivities exhibited by E/GEJ PTXG models exposed to standard therapies. A wide spectrum of patient response to identical therapy is a major clinical challenge. PTXG models recapitulated this finding, by exhibiting a wide range of sensitivities and resistances to standard chemotherapy, by developing resistance upon repeated treatment (i.e., Line F). Although limited by small sample size, our patient drug responses matched PTXG responses, a finding consistent with another study involving many primary cancer sites [17]. Stability of drug sensitivity results across passages is another indication of a useful model.

As a limitation, our study was not designed to have adequate power to identify or correlate with markers of chemosensitivity; rather, understanding the utility and limitations of the model is a critical step before creating additional models to address these other important questions. In contrast to the tumor growth process seen in humans, immune response in the xenograft models is lacking. The role of immunity and inflammation is especially important for esophageal adenocarcinomas where gastric reflux has shown to promote tumorigenesis through chronic inflammation [34]. Unfortunately, the effects that immunosuppression has on tumor development and treatment cannot be replicated using these models, and will limit usefulness of the model when immunotherapy is being evaluated.

In conclusion, we demonstrated that primary esophageal cancer xenografts are useful and stable models for drug testing, with known biases (implantation/engraftment selection) and confounding by known factors (intra- and inter-tumor heterogeneity) that can be addressed, and make the model more similar to primary human tumors. There was a wide range of responses to standard chemotherapy, consistent with what is seen in the clinical setting. Overall, these models are potentially useful in the evaluation of novel therapeutics and their combinations.

## Supporting Information

S1 Fig Unsupervised hierarchical clustering of normalized signal intensities of all genes from xenografts and patient samples, showing intra-array correlations. Color bars shown indicate (A) patient and xenograft samples, (B) male and female samples, (C) array batches and (D) Tumor lines involved in experiments (white: samples not involved in any other comparisons). Analysis shows that xenografts cluster together with other xenografts (similarly patient tumors cluster together) and that samples cluster based on their tumor line (*i*.*e*. all xenografts of the same line cluster together).
(TIF)

S2 Fig Distribution of the delay at doubling time for the different lines treated with chemotherapeutics. According to this distribution, the lines can be divided into three different categories. The resistant lines (A and B) show very little delay at the doubling time (average of 2.3 days). Lines C and D show intermediate chemosensitivity to the drug combination (average of 4.4 days of delay at doubling time). After treatment, these lines were characterized by a decrease in the average tumor volume (labeled as “trough”) followed by a regrowth period with a similar slope to the control. Lastly, lines E, F and G show the highest level of sensitivity to the drug combination with an average of 6.7 days of delay at doubling time. These lines were characterized by a shallower slope, indicating a reduced rate of growth (labeled as “Δ Slope”) when compared to the control group.
(TIF)

S3 Fig Chemosensitivity of xenograft lines in different passages. Line B showed clear chemoresistance towards individual drugs and when treated with a combined dose of cisplatin and paclitaxel. When treated with an initial dose of individual chemotherapeutics, Line F exhibited initial chemosensitivity but when exposed to a second dose of drugs it showed chemoresistance. A similar scenario occurred when Line F was treated with a combined dose of cisplatin and paclitaxel (at first it was sensitive and then it acquired resistance). The black arrow shows the first treatment time while the green arrow shows the second treatment time. Chemosensitivity to a combined dose of paclitaxel and cisplatin did not change between consecutive passages (P4 to P5) of Line D.
(TIF)

S1 Table Univariate Analysis of Clinicopathological Characteristics of All Primary Human Tumors. (DOCX)

S2 Table Univariate Analysis of Clinicopathological Characteristics of Primary Human Adenocarcinomas. (DOCX)
