# Supplementary figures and images for: Appropriateness of Using Patient-Derived Xenograft Models for Pharmacologic Evaluation of Novel Therapies for Esophageal/Gastro-Esophageal Junction Cancers
Source: PLoS One. 2015 Mar 31;10(3):e0121872. doi: 10.1371/journal.pone.0121872 (PMC4380353; doi:10.1371/journal.pone.0121872)

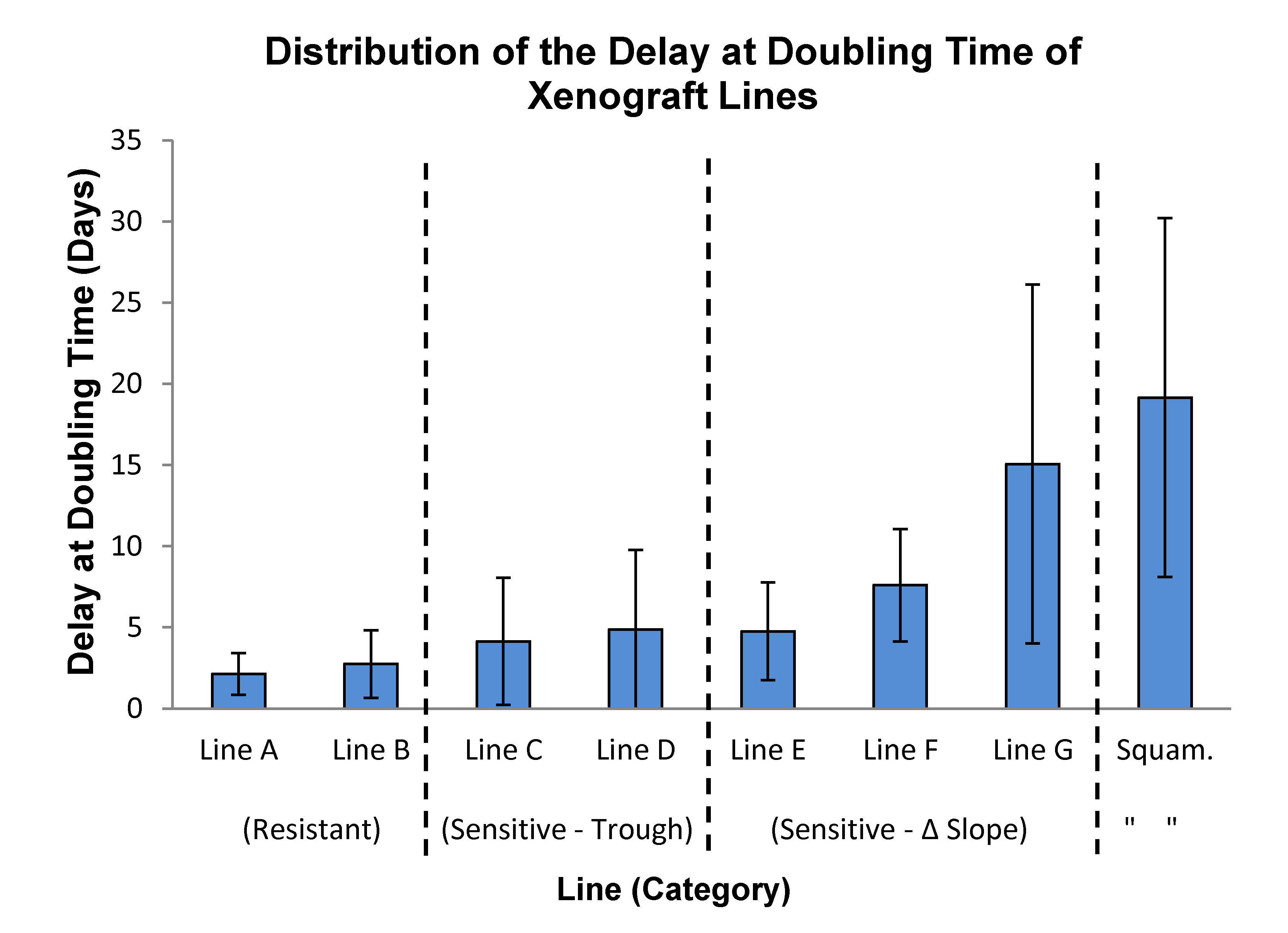

Supplement: S2 Fig — According to this distribution, the lines can be divided into three different categories. The resistant lines (A and B) show very little delay at the doubling time (average of 2.3 days). Lines C and D show intermediate chemosensitivity to the drug combination (average of 4.4 days of delay at doubling time). After treatment, these lines were characterized by a decrease in the average tumor volume (labeled as “trough”) followed by a regrowth period with a similar slope to the control. Lastly, lines E, F and G show the highest level of sensitivity to the drug combination with an average of 6.7 days of delay at doubling time. These lines were characterized by a shallower slope, indicating a reduced rate of growth (labeled as “Δ Slope”) when compared to the control group. (TIF) [file pone.0121872.s002.tif]

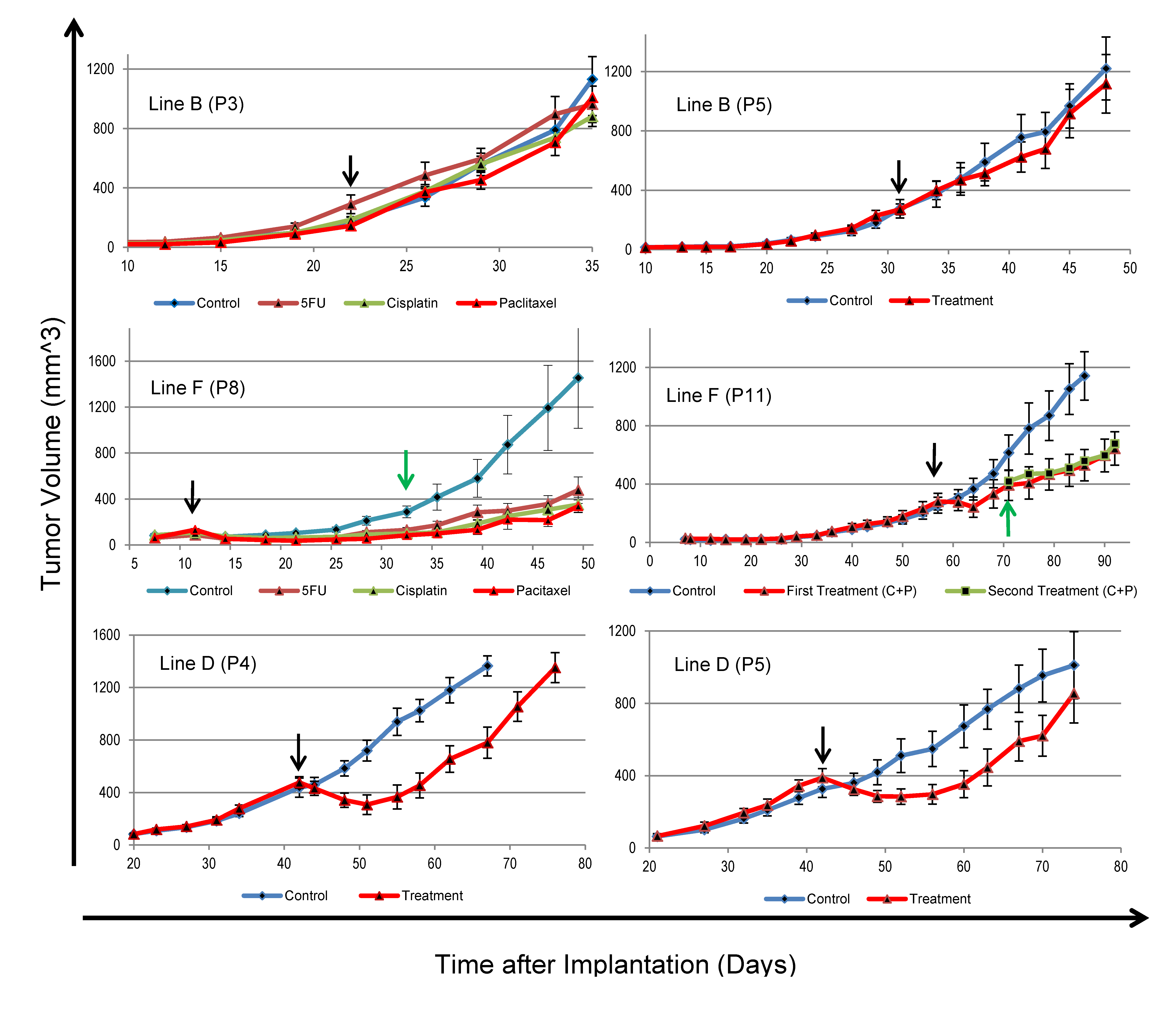

Supplement: S3 Fig — Line B showed clear chemoresistance towards individual drugs and when treated with a combined dose of cisplatin and paclitaxel. When treated with an initial dose of individual chemotherapeutics, Line F exhibited initial chemosensitivity but when exposed to a second dose of drugs it showed chemoresistance. A similar scenario occurred when Line F was treated with a combined dose of cisplatin and paclitaxel (at first it was sensitive and then it acquired resistance). The black arrow shows the first treatment time while the green arrow shows the second treatment time. Chemosensitivity to a combined dose of paclitaxel and cisplatin did not change between consecutive passages (P4 to P5) of Line D. (TIF) [file pone.0121872.s003.tif]
