# Supplementary material for: Appropriateness of Using Patient-Derived Xenograft Models for Pharmacologic Evaluation of Novel Therapies for Esophageal/Gastro-Esophageal Junction Cancers
Source: PLoS One. 2015 Mar 31;10(3):e0121872. doi: 10.1371/journal.pone.0121872 (PMC4380353; doi:10.1371/journal.pone.0121872)
Supplement: S1 Table — (DOCX) [file pone.0121872.s004.docx]

| **Supplementary Table S1 –** Univariate Analysis of Clinicopathological Characteristics of All Primary Human Tumors | | | | | | | | | | | | | | |
| --- | --- | --- | --- | --- | --- | --- | --- | --- | --- | --- | --- | --- | --- | --- |
| **All Esophageal Cancer Subtypes** | | **Implanted (I) *vs* Non-Implanted (NI)** | | | | | **Engrafted (E) *vs* Non-Engrafted (NE)** | | | | **Engrafted (E) *vs* Others (O)** | | | |
| **Characteristic** | **Comparison** | **NI (32)** | **I (55)** | **OR (95% CI)** | | **p-value** | **NE (34)** | **E (21)** | **OR (95% CI)** | **p-value** | **O (66)** | **E (21)** | **OR(95% CI)** | **p-value** |
| Age (years) | Per 10 Year Incr. | n/a | | 0.99 (0.95,1.03) | | 0.57 | n/a | | **1.08 (1.02,1.14)** | **0.01** | n/a | | **1.06 (1,1.11)** | **0.03** |
| Gender | M | 27 | 42 | Reference | | 0.38 | 28 | 14 | Reference | 0.19 | 55 | 14 | Reference | 0.11 |
|  | F | 5 | 13 | 1.67 (0.53,5.22) | |  | 6 | 7 | 2.33 (0.66,8.27) |  | 11 | 7 | 2.5 (0.82,7.62) |  |
| Stage^a^ | I/II | 17 | 25 | Reference | | 0.22 | 17 | 8 | Reference | 0.48 | 34 | 8 | Reference | 0.25 |
|  | III/IV | 11 | 29 | 1.79 (0.71,4.53) | |  | 17 | 12 | 1.5 (0.49,4.59) |  | 28 | 12 | 1.82 (0.65,5.08) |  |
| Differentiation^a^ | Mod./Well | 20 | 33 | Reference | | 0.98 | 25 | 8 | Reference | **0.01** | 45 | 8 | Reference | **0.02** |
|  | Poorly | 12 | 20 | 1.01 (0.41,2.5) | |  | 8 | 12 | **4.69 (1.42,15.53)** |  | 20 | 12 | **3.37 (1.2,9.53)** |  |
| Location | GE Junction | 10 | 34 | **Reference** | | **0.03** | 21 | 13 | Reference | 0.78 | 31 | 13 | Reference | 0.45 |
|  | Lower Third/Distal | 15 | 15 | 0.29 (0.11,0.8) | |  | 10 | 5 | 0.81 (0.23,2.9) |  | 25 | 5 | 0.48 (0.15,1.52) |  |
|  | Mid/Upper | 7 | 6 | 0.25 (0.07,0.92) | |  | 3 | 3 | 1.62 (0.28,9.23) |  | 10 | 3 | 0.72 (0.17,3.03) |  |
| Neo-adjuvant Chemo-Rad | No | 31 | 33 | Reference | | **0.004** | 17 | 16 | Reference | 0.06 | 48 | 16 | Reference | 0.75 |
|  | Yes | 1 | 22 | **20.67 (2.63,162.65)** | |  | 17 | 5 | 0.31 (0.09,1.05) |  | 18 | 5 | 0.83 (0.27,2.61) |  |
| Heartburn | No or n/a | 22 | 29 | Reference | | 0.14 | 19 | 10 | Reference | 0.55 | 41 | 10 | Reference | 0.24 |
|  | Yes | 10 | 26 | 1.97 (0.79,4.93) | |  | 15 | 11 | 1.39 (0.47,4.15) |  | 25 | 11 | 1.8 (0.67,4.86) |  |
| Barrett's Esophagus | No or n/a | 25 | 45 | Reference | | 0.67 | 27 | 18 | Reference | 0.56 | 52 | 18 | Reference | 0.49 |
|  | Yes | 7 | 10 | 0.79 (0.27,2.34) | |  | 7 | 3 | 0.64 (0.15,2.82) |  | 14 | 3 | 0.62 (0.16,2.41) |  |
| **Immunohistochemical Characteristics of All Primary Human Tumors** | | | | | | | | | | | | | | |
| **All Esophageal Cancer Subtypes** | | **Implanted (I) *vs* Non-Implanted (NI)** | | | | | **Engrafted (E) *vs* Non-Engrafted (NE)** | | | | **Engrafted (E) *vs* Others (O)** | | | |
| **Characteristic** | **Variable** | **NI (32)** | **I (55)** | **OR (95% CI)** | **p-value** | | **NE (34)** | **E (21)** | **OR (95% CI)** | **p-value** | **O (66)** | **E (21)** | **OR(95% CI)** | **p-value** |
| Her-2/*neu* | Per 1 Stain Incr. | n/a | | 1.35 (0.86,2.52) | 0.16 | | n/a | | 1.31 (0.76,2.26) | 0.33 | n/a | | 1.46 (0.87,2.43) | 0.15 |
| EGFR | Per 1 Stain Incr. | n/a | | 0.7 (0.47,1.04) | 0.08 | | n/a | | 0.99 (0.58,1.7) | 0.97 | n/a | | 0.82 (0.5,1.33) | 0.42 |
| Ki-67  (% positive) | 20% unit increase | n/a | | 1.23 (0.85,1.78) | 0.26 | | n/a | | 1.2 (0.77,1.85) | 0.42 | n/a | | 1.29 (0.86,1.93) | 0.22 |
| p16 | Negative | 22 | 37 | Reference | 0.87 | | 20 | 17 | Reference | 0.10 | 42 | 17 | Reference | 0.15 |
|  | Positive | 10 | 18 | 1.07 (0.42,2.73) |  |  | 14 | 4 | 0.34 (0.09,1.22) |  | 24 | 4 | 0.41 (0.12,1.37) |  |
| p53 | Negative | 18 | 25 | Reference | 0.33 | | 16 | 9 | Reference | 0.76 | 34 | 9 | Reference | 0.49 |
|  | Positive | 14 | 30 | 1.54 (0.64,3.71) |  |  | 18 | 12 | 1.19 (0.4,3.55) |  | 32 | 12 | 1.42 (0.53,3.81) |  |
| ^a^ Patients with no available information were not included in the calculation. | | | | | | | | | | | | | | |
