# Supplementary material for: Appropriateness of Using Patient-Derived Xenograft Models for Pharmacologic Evaluation of Novel Therapies for Esophageal/Gastro-Esophageal Junction Cancers
Source: PLoS One. 2015 Mar 31;10(3):e0121872. doi: 10.1371/journal.pone.0121872 (PMC4380353; doi:10.1371/journal.pone.0121872)
Supplement: S2 Table — (DOCX) [file pone.0121872.s005.docx]

| **Supplementary Table S2 –** Univariate Analysis of Clinicopathological Characteristics of Primary Human Adenocarcinomas | | | | | | | | | | | | | |
| --- | --- | --- | --- | --- | --- | --- | --- | --- | --- | --- | --- | --- | --- |
| **Adenocarcinomas** | | **Implanted (I) *vs* Non-Implanted (NI)** | | | | **Engrafted (E) *vs* Non-Engrafted (NE)** | | | | **Engrafted (E) *vs* Others (O)** | | | |
| **Characteristic** | **Level** | **NI (22)** | **I (43)** | **OR (95% CI)** | **p-value** | **NE (26)** | **E (17)** | **OR (95% CI)** | **p-value** | **O (48)** | **E (17)** | **OR (95% CI)** | **p-value** |
| Age (years) | Per 10 Year Incr. | n/a | | 0.99 (0.95,1.04) | p=0.72 | n/a | | 1.06 (1,1.13) | p=0.0504 | n/a | | 1.04 (0.99,1.1) | p=0.10 |
| Gender | M | 21 | 34 | Reference | p=0.12 | 21 | 13 | Reference | p=0.74 | 42 | 13 | Reference | p=0.29 |
|  | F | 1 | 9 | 5.56 (0.66,47.08) |  | 5 | 4 | 1.29 (0.29,5.71) |  | 6 | 4 | 2.15 (0.53,8.82) |  |
| Stage* | I/II | 10 | 18 | Reference | p=0.72 | 12 | 6 | Reference | p=0.58 | 22 | 6 | Reference | p=0.52 |
|  | III/IV | 11 | 24 | 1.21 (0.42,4.47) |  | 14 | 10 | 1.43 (0.4,5.1) |  | 25 | 10 | 1.47 (0.46,4.69) |  |
| Differentiation* | Mod./Well | 13 | 24 | Reference | p=0.97 | 19 | 5 | Reference | **p=0.01** | 32 | 5 | Reference | **p=0.01** |
|  | Poorly | 9 | 17 | 1.02 (0.36,2.93) |  | 6 | 11 | **6.97 (1.72,28.25)** |  | 15 | 11 | **4.69 (1.38,15.93)** |  |
| Location | GE Junction | 10 | 33 | **Reference** | **p=0.01** | 20 | 13 | Reference | p=0.97 | 30 | 13 | Reference | p=0.30 |
|  | Lower Third/Distal | 12 | 10 | 0.25 (0.08,0.76) |  | 6 | 4 | 1.03 (0.24,4.35) |  | 18 | 4 | 0.51 (0.14,1.81) |  |
| Neoadjuvant Chemo-Rad | No | 22 | 23 | Reference | **p=0.004** | 10 | 13 | Reference | **p=0.02** | 32 | 13 | Reference | p=0.45 |
|  | Yes | 0 | 20 | **-** |  | 16 | 4 | **0.19 (0.05,0.76)** |  | 16 | 4 | 0.62 (0.17,2.19) |  |
| Heartburn | No or n/a | 16 | 24 | Reference | p=0.19 | 15 | 9 | Reference | p=0.76 | 31 | 9 | Reference | p=0.40 |
|  | Yes | 6 | 19 | 2.11 (0.69,6.44) |  | 11 | 8 | 1.21 (0.35,4.15) |  | 17 | 8 | 1.62 (0.53,4.97) |  |
| Barrett's Esophagus | No or n/a | 15 | 33 | Reference | p=0.46 | 19 | 14 | Reference | p=0.48 | 34 | 14 | Reference | p=0.36 |
|  | Yes | 7 | 10 | 0.65 (0.21,2.04) |  | 7 | 3 | 0.58 (0.13,2.66) |  | 14 | 3 | 0.52 (0.13,2.1) |  |
| **Immunohistochemical Characteristics of Primary Human Adenocarcinomas** | | | | | | | | | | | | | |
| **Adenocarcinomas** | | **Implanted (I) *vs* Non-Implanted (NI)** | | | | **Engrafted (E) *vs* Non-Engrafted (NE)** | | | | **Engrafted (E) *vs* Others (O)** | | | |
| **Characteristic** | **Level** | **NI (25)** | **I (42)** | **OR (95% CI)** | **p-value** | **NE**  **(27)** | **E (15)** | **OR (95% CI)** | **p-value** | **O (52)** | **E (15)** | **OR (95% CI)** | **p-value** |
| Her2-Neu | Per 1 Stain Incr. | n/a | | 1.32 (0.75,2.32) | p=0.34 | n/a | | 1.32 (0.74,2.36) | p=0.35 | n/a | | 1.44 (0.83,2.51) | p=0.19 |
| EGFR | Per 1 Stain Incr. | n/a | | 0.85 (0.41,1.77) | p=0.66 | n/a | | 1.17 (0.5,2.71) | p=0.71 | n/a | | 1.07 (0.48,2.37) | p=0.87 |
| Ki-67  (% positive) | 20% unit increase | n/a | | 1.16 (0.77,1.76) | p=0.47 | n/a | | 1.17 (0.73,1.87) | p=0.52 | n/a | | 1.22 (0.79,1.89) | p=0.36 |
| p16 | Negative | 15 | 31 | Reference | p=0.74 | 17 | 14 | Reference | p=0.23 | 32 | 14 | Reference | p=0.23 |
|  | Positive | 7 | 12 | 0.83 (0.27,2.54) |  | 9 | 3 | 0.4 (0.09,1.79) |  | 16 | 3 | 0.43 (0.11,1.71) |  |
| p53 | Negative | 12 | 22 | Reference | p=0.8 | 13 | 9 | Reference | p=0.85 | 25 | 9 | Reference | p=0.95 |
|  | Positive | 10 | 21 | 1.15 (0.41,3.21) |  | 13 | 8 | 0.89 (0.26,3.02) |  | 23 | 8 | 0.97 (0.3,2.93) |  |
| * Patients with no available information were not included in the calculation. | | | | | | | | | | | | | |
